# Supplementary material for: Identification of photoperiod-induced specific miRNAs in the adrenal glands of Sunite sheep (Ovis aries)
Source: Front Vet Sci. 2022 Jul 22;9:888207. doi: 10.3389/fvets.2022.888207 (PMC9354845; doi:10.3389/fvets.2022.888207)
Supplement: Supplementary file 1 [file Data_Sheet_1.ZIP › Supplementary file/miRDeep2 file.zip!/miRDeep2 file/Novel_Structure/LP42A1/Novel_220.pdf]

Provisional ID	:	NC_019467.2_17541
Score total	:	1.6
Score for star read(s)	:	-1.3
Score for read counts	:	0
Score for mfe	:	1.3
Score for randfold	:	1.6
Score for cons. seed	:	
Total read count	:	487
Mature read count	:	487
Loop read count	:	0
Star read count	:	0

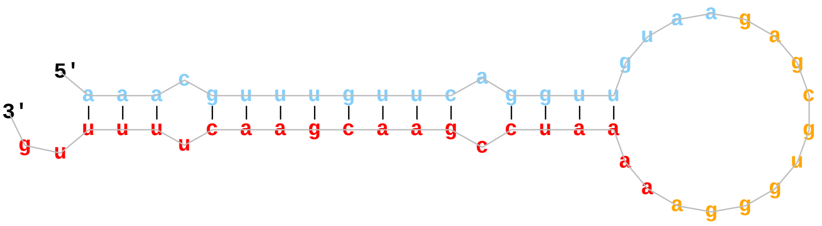

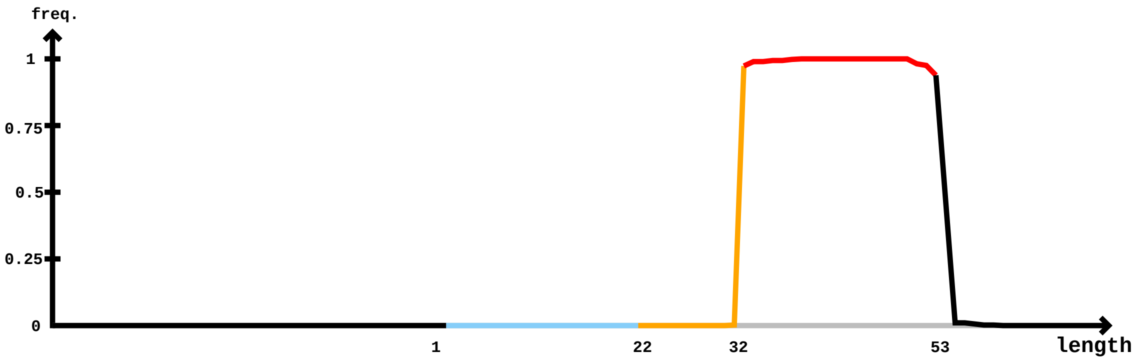

Star Mature

```
5'- uggguucuguccuagguaaugguaguacuggguuggccaaaacguuuguucagguuguaagagcgugggaaaaauccgaacgaacuuuuuggccaagucaauaauuug   -3'    exp
    ..(((.(((.((........))))).)))((.((((((((((.((((((((.((((................)))).)))))))).)))))))))).)).........   reads     mm         sample
    .....................................................................aaaaauccgaaUgaacuuuuu..................   1         1          seq
    ......................................................................aaaauccgaacgaacuuu....................   9         0          seq
    ......................................................................aaaaucUgaacgaacuuuuu..................   8         1          seq
    ......................................................................aaaauccgaacAaacuuuuu..................   6         1          seq
    ......................................................................aaaauccAaacgaacuuuuu..................   3         1          seq
    ......................................................................aaaauccgaacgaacuuuuug.................   443       0          seq
    ......................................................................aaaauccAaacgaacuuuuug.................   1         1          seq
    ......................................................................aaaauccAaacgaacuuuuugg................   2         1          seq
    ......................................................................aaaaucUgaacgaacuuuuugg................   2         1          seq
    .......................................................................aaauccgaacgaacuuu....................   3         0          seq
    .......................................................................aaauccgaacgaacuuuuugg................   5         0          seq
    .........................................................................auccgaacAaacuuuuuggcc..............   2         1          seq
    ...........................................................................ccgaacgaacuuuuuggcca.............   1         0          seq
    ...........................................................................ccgaacgaacuuuuuggccaaU...........   1         1          seq
    ............................................................................cgaacgaacuuuuuggcca.............   1         0          seq
```
